# Cytotoxicity of curcumin against CD44^±^ prostate cancer cells: Roles of miR-383 and miR-708

**DOI:** 10.22038/AJP.2023.21913

**Published:** 2023

**Authors:** Reza Panahizadeh, Mohammad Amin Vatankhah, Farhad Jeddi, AmirAhmad Arabzadeh, Kazem Nejati-Koshki, Ramin Salimnejad, Nowruz Najafzadeh

**Affiliations:** 1 *Students Research Committee, School of Medicine, Ardabil University of Medical Sciences, Ardabil, Iran*; 2 *Research laboratory for Embryology and Stem Cells, Department of Anatomical Sciences, School of Medicine, Ardabil University of Medical Sciences, Ardabil, Iran *; 3 *Department of Medical Genetics and Pathology, Faculty of Medicine, Ardabil University of Medical Sciences, Ardabil, Iran *; 4 *Department of Surgery, School of Medicine, Ardabil University of Medical Sciences, Ardabil, Iran *; 5 *Pharmaceutical Sciences Research Center, Ardabil University of Medical Science, Ardabil, Iran *

**Keywords:** Prostate neoplasms, MicroRNAs, hsa-miR-708-5p, hsa-miR-383-5p, Natural products, Curcumin

## Abstract

**Objective:**

Cancer stem cells (CSCs) remaining in the tumor tissues after applying treatments may cause recurrence or metastasis of prostate cancer (PC). Curcumin has the promising potential to target CSCs. Here, we aim to evaluate the cytotoxic effects of curcumin on the expression of miR-383-5p and miR-708-5p and their target genes in CD44^+^ CSCs and CD44^-^ non-CSCs isolated from the PC3 prostate cancer cell line.

**Materials and Methods:**

We used MTT assay to determine the optimal cytotoxic dose of curcumin on CD44^± ^PC cells. Then, we assessed nuclear morphological changes using DAPi staining. We used Annexin V-FITC/PI to quantify apoptotic cell death. qRT-PCR was also used to detect miRNA and gene expression levels after curcumin treatment.

**Results:**

Curcumin significantly enhanced the apoptosis in both CD44^-^ and CD44^+^ PC cells in a dose-dependent manner (p < 0.05). The cytotoxicity of curcumin against CD44^-^ cells (IC_50 _ 40.30±2.32 μM) was found to be greater than that against CD44^+^ cells (IC_50 _ 83.31±2.91 μM). Also, curcumin promoted miR-383-5p and miR-708-5p overexpression while downregulating their target genes *LDHA*, *PRDX3*, and *RAP1B*, *LSD1*, respectively.

**Conclusion:**

Our findings indicate that curcumin, by promoting the expression of tumor suppressors, miR-383-5p and miR-708-5p, and inhibiting their target genes, induced its cytotoxicity against CD44^±^ PC cells. We trust that curcumin could be established as a promising adjuvant therapy to current PC treatment options following more research in clinical settings.

## Introduction

According to GLOBOCAN 2020, prostate cancer (PC) incidence is almost 1.3 million per year. In 2020, PC accounted for 359,000 deaths worldwide (Sung et al., 2021). A more and deeper understanding of PC's pathogenesis and molecular basis is critical to improving treatments (Karayi and Markham, 2004; Moshtaghioun et al., 2021). It is estimated that genetic factors play a critical role in 40% of early-onset PCs and 5-10% of all PCs (Beebe-Dimmer et al., 2020). The prognosis of patients diagnosed with localized lesions of PC is good, but the survival rate of metastatic cases drops down to 31%. Radical surgery, radiation therapy, cryotherapy, and androgen deprivation therapy are commonly used to treat PC according to the stage of the tumors. Although applying appropriate treatments abolish the main part of the tumor, cancer stem cells (CSCs) remaining in the tumor tissues may cause recurrence or metastasis of the tumor (Harris and Kerr, 2017). CSCs, a small subset of tumor cells, have the ability for self-renewal and asymmetric division, making them less vulnerable to chemotherapy and radiotherapy. So, the reason behind the failure of chemotherapy is the inability of current chemotherapeutics to eliminate CSCs (DeSano and Xu, 2009). Several markers such as CD44, CD133, and ABCG2 have been introduced to identify prostate CSCs. CD44 is one of the main prostate CSC tumor markers, and its high expression has been associated with tumor proliferation, stemness gene expression, and metastatic colonization (Harris and Kerr, 2017).

microRNAs (miRNAs) are small non-coding RNAs that alter gene expression by post-transcriptional silencing of the genes (Mokabber et al., 2022). The first correlation between miRNAs and cancer was discovered over two decades ago. Since then, miRNAs have been investigated in almost all cancer types. Ingenious technological developments for the detection of miRNAs in tissue and bodily fluids, the identification of the diagnostic and prognostic value of specific miRNAs, and the development of numerous miRNA delivery strategies for therapeutic intervention have all been made possible by this incredibly large amount of research (Nejati et al., 2021; Pahlavan et al., 2020; Sempere et al., 2021). miRNAs such as miR-708-5p and miR-383-5p have been shown to be remarkably downregulated in PC (Bucay et al., 2017; Saini et al., 2012). Recent studies have approved that CD44 is a direct target of miR-708 and miR-383. miR-708 is a negative regulator of CD44^+^ prostate CSCs, which can be used for prognosis and diagnosis of PC. Induction of ectopic expression of miR-383 has inhibited the tumorigenesis process and metastasis in CD44^+ ^prostate CSCs. Downregulation of miR-708 and miR-383 has been correlated with poor prognosis, tumor growth, and recurrence in clinical specimens. Together, overexpressing mir-708 and miR-383 could serve as an excellent attempt to treat metastatic PC (Bucay et al., 2017; Saini et al., 2012).

Natural products are valuable resources that have been frequently used to discover and produce anti-cancer drugs (Bishayee and Sethi, 2016). Recent studies on plant extracts of tomato, soy, ginger, and garlic have shown that bioactive molecules derived from these extracts could diminish chemoresistance and tumor growth (Grainger et al., 2019; Liu et al., 2017a; Salehi et al., 2019; Samy et al., 2021). Curcumin or diferuloylmethane is a bioactive metabolite derived from the *Curcuma longa *L. plant, primarily used in Chinese traditional medicine. Curcumin is a phytochemical product whose anti-PC effects on the inhibition of proliferation, invasion, cellular adhesion, and angiogenesis of many tumors have been confirmed (Termini et al., 2020). Multiple studies have shown that curcumin and its analogs have promising potential to target CSCs (Li and Zhang, 2014; Ramasamy et al., 2015). However, studies suggest that curcumin causes anti-cancer effects by regulating miRNAs and their downstream molecular pathways (Mirzaei et al., 2018; Zendehdel et al., 2019), the role of curcumin in the regulation of miR-708 and miR-383 expression has not been elucidated yet.

According to these facts, our objective is to assess the molecular mechanism of cytotoxicity of curcumin and the efficacy of curcumin on the regulation of miR-708-5p, miR-383-5p, and their target genes in prostate CSCs and non-CSCs.

## Materials and Methods

**Drugs and reagents**

Curcumin (C1386) and MTT (3-(4,5-dimethylthiazol-2-yl)-2, 5-diphenyl tetrazolium bromide, M2128) were obtained from Sigma-Aldrich (St. Louis, MO, USA). Curcumin was dissolved in dimethylsulfoxide (DMSO) and stored at -20ºC.

**Cell culture**

The human prostate cancer cell line, PC3, was obtained from the National Cell Bank of Iran (NCBI, Pasteur Institute, Tehran, Iran). Cells were grown in RPMI 1640 medium (Gibco, UK), containing 10 % fetal bovine serum (FBS, Gibco), penicillin, and streptomycin. Finally, the cells were incubated in a humidified atmosphere of 5% CO_2_ at 37ºC.

**Isolation of CD44** ^±^ **cells by magnetic-activated cell sorting (MACS)**

CD44- positive CSCs and CD44- negative non-CSCs were sorted using MACS. Concisely, PC3 prostate cancer cells were trypsinized and collected. Then, 10^6^ cells were stained with anti-human CD44 primary antibody (Miltenyi Biotec, 130-095-180). After incubating the cell suspension with Anti-PE Micro Bead (Miltenyi Biotec,130-048-801), the cells were passed through a MACS column within a magnetic field. CD44^+^ cells carrying the magnetic beads were retained inside the column and adsorbed onto the surface. We sorted the cells twice to achieve more than 95% pure cells. 

**MTT assay**

Concisely, the CD44^±^ prostate cancer cells were seeded in 96-well plates. The CD44^-^ cells were incubated in an RPMI1640 medium containing 0-70 µM curcumin. The CD44^+^ prostate cancer cells were treated with 15-150 µM curcumin. Curcumin dosage was determined according to similar research and our previous studies (Mollazade et al., 2013; Nejati-Koshki et al., 2014; Vatankhah et al., 2022). After 48 hr of incubation, the medium was replaced with 180 ml FBS-free medium and 20 ml MTT (Sigma, M2128). Then the cells were incubated for 4 hr at 37ºC. Then, 200 ml DMSO (Scharlau Chemie, and Barcelona, Spain) was used to dissolve formazan crystals. Finally, the absorbance was measured at 570 nm using an ELISA reader. 

**Apoptosis assessment**

The cytotoxicity and nuclear morphological changes induced by curcumin were examined by DAPi staining. Briefly, the CD44^±^ cells (5 × 10^4^ cells/well) were seeded into 6-well plates. The CD44^-^ and CD44^+^ cells were treated with 15-60 µM and 50-150 µM curcumin, respectively. Curcumin dosage was determined according to similar research and our previous studies (Mollazade et al., 2013; Nasiri et al., 2013; Nejati-Koshki et al., 2014; Vatankhah et al., 2022). Then, the cells were fixed and stained with DAPi (Fatehi-Agdam et al., 2021). 

**Quantification of gene expression by real-time PCR**

Total RNA was extracted from CD44^± ^prostate cancer cells using TRIzol reagent (Invitrogen). The total RNA was reverse-transcribed into cDNA using a Viva 2-steps RT-PCR kit (Vivantis, USA). Briefly, we used SYBR Green PCR Master Mix (EURx, Ltd, Poland) to perform qRT-PCR, and gene expression was analyzed using a Light Cycler 96 real-time PCR System (Roche Applied Science) (Fatehi-Agdam et al., 2021; Khakbaz et al., 2021). The PCR reaction was conducted in a total volume of 20 μl containing 10 μl of master mix, 4 μl of cDNA, and 2 μl of primer. Fold changes in gene expression were calculated using the ΔΔCt method relative to *GAPDH* gene expression. The primers are listed in Table 1.

**Quantitation of microRNAs by real-time PCR**

The expression levels of miR-383‐5p and miR-708-5p were measured using BON-miR miRNA cDNA Synthesis Kit (Cat: BON209001). The following primers were used in qPCR reactions: miR‐383‐5p (TAGATCAGAAGGTGATTG), miR-708-5p (GGAAGGAGCTTACAATCTA), and U6 (AAGGATGACACGCAAA). Briefly, after polyadenylation, a PCR reaction was performed in a 10 µl reaction containing 1.0 μl reverse transcriptase (RT) enzyme, 10 μM Bon‐RT primer, 100 mM dNTP mix, and 2.0 μl 10 × RT buffer. miRNA expression fold changes were evaluated using the comparative ∆∆Ct method (Mokabber et al., 2019).

**Flow cytometry**

Human CD44^±^ prostate cancer cells were cultivated at a density of 5×10^5 ^cells/well in a 6-well plate. The CD44^±^ cells were treated with subtoxic concertation of curcumin for 48 hr. The cells were counterstained with Annexin V-FITC (fluorescein isothiocyanate)/PI (propidium iodide) (IQ products, Netherlands) staining assay. Briefly, 1×10^6^ cells were centrifuged and rinsed with calcium-binding buffer, and 5 µl of Annexin V/FITC was added to the cell suspension. Finally, analysis was performed by a flow cytometer (Partec CyFlow®) with emission filters of 515–545 nm for FITC (green) and 600 nm for PI (red) (Rahnamay et al., 2018).

**Statistical analysis**

Data analysis was performed using SPSS V.21 software. One-way ANOVA with Tukey’s *post hoc* test was used for statistical comparisons. A p-value less than 0.05 was considered significant. The standard curves were drawn using sigma plot V.11 software (Systat Software, San Jose, CA).

## Results

**Curcumin induces cytotoxicity against CD44** ^-^ **cells and CD44** ^+^ **prostate cancer cells.**

**Figure 1:**
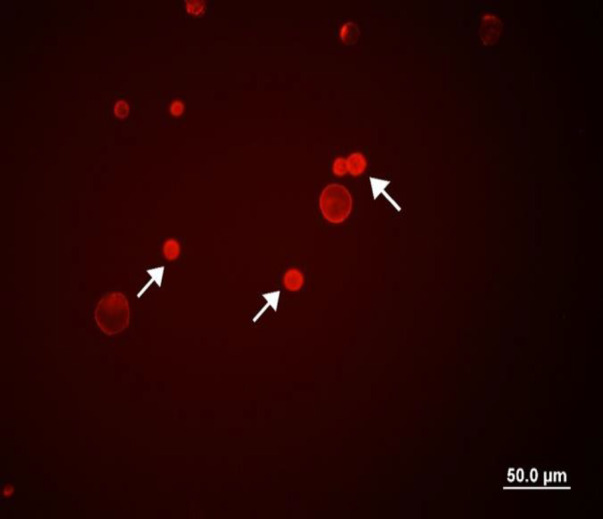
Isolation of CD44^±^ cells from PC3 cell line using MACS. CD44^+^ cells were isolated and identified by fluorescence microscopy. The arrows show CD44^+^ prostate CSCs.

CD44^+^ PC3 cells are shown in Figure 1. The cell survival rate was determined using the MTT cell proliferation assay. The IC_50_ values of curcumin for CD44^-^ and CD44^+^ prostate cancer cells were 40.30±2.32 μM and 83.31±2.91 µM, respectively. The IC_50_ value of curcumin against CD44^-^cells was significantly lower than that observed for CD44^+^ cells (p<0.05).

**Figure 2:**
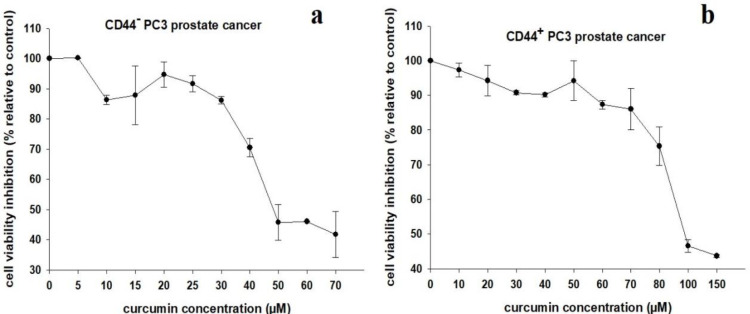
Standard curve of the viability of CD44^± ^PC3 cells treated with different concentrations of curcumin.

Our results confirmed that curcumin inhibited both CD44^-^ and CD44^+^ prostate cancer cells in a dose-dependent manner. The cytotoxicity of curcumin against CD44^-^ cells was more greater than that found for CD44^+^ cells. Indeed, CD44^+^ cells were more resistant to curcumin treatment (Figure 2).

**The role of curcumin treatment on the morphology and apoptosis**

**Figure 3:**
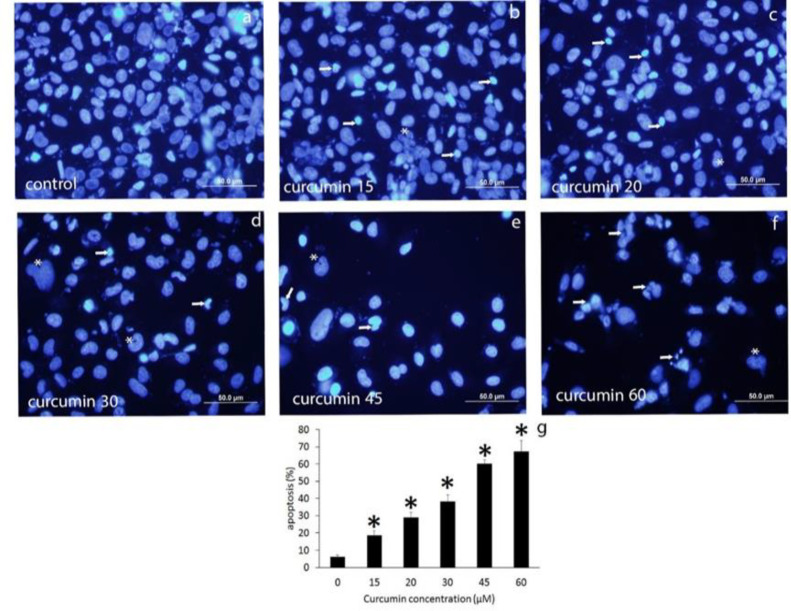
DAPi staining of CD44^-^ PC3 cells treated with 15 – 60 µM curcumin. Apoptotic cells are shown with arrows. Autophagic cells are marked with asterisks (a-g). *p<0.05 vs. control

**Figure 4:**
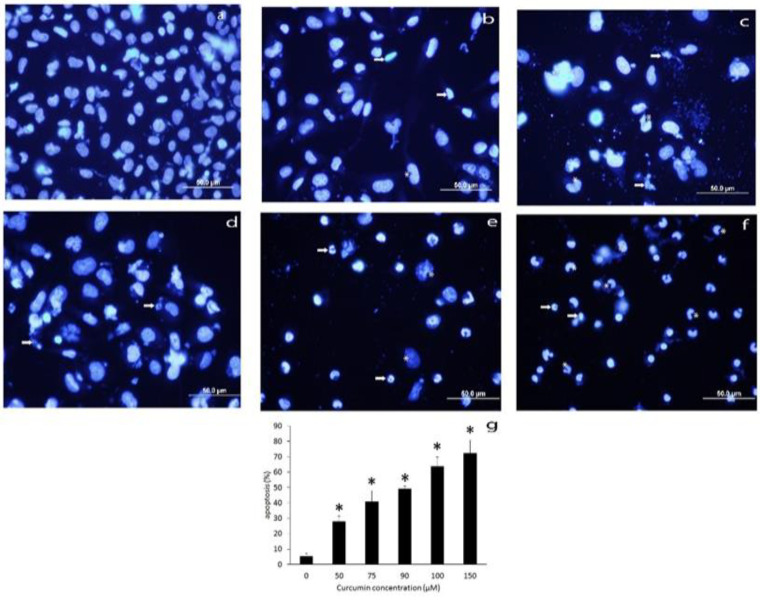
DAPi staining of CD44^+^ PC3 cells treated with 50 – 150 µM curcumin. Apoptotic bodies are shown with arrows and autophagic cells are marked with asterisks (a-g). *p<0.05 vs. control

Nuclear shrinkage, chromatin condensation, and pyknotic body formation were observed in response to the treatment with curcumin. Autophagic cell morphology was also seen after treatment with high doses of curcumin in CD44^±^ PC3 cells (Figure 3 and Figure 4).

**Figure 5:**
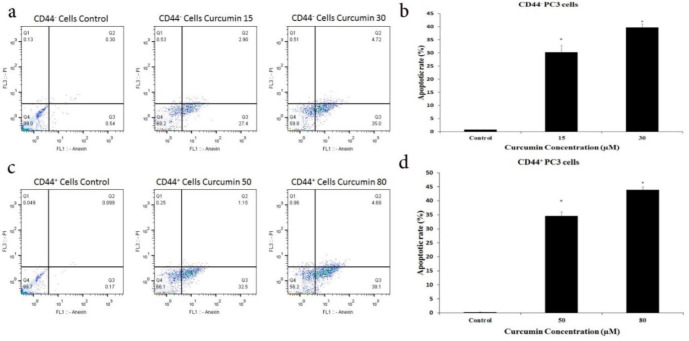
Effects of curcumin on the apoptosis of CD44^± ^PC3 cells. (A, C) Flow cytometric analysis and (B, D) apoptotic rates of CD44^± ^PC3 cells treated with subtoxic concentrations of curcumin for 48 hr. Live cells: Q4, Apoptosis: Q2 and Q3, Necrotic cells: Q1. *p <0.001 vs. control. PI, propidium iodide

Indeed, flow cytometric results revealed that curcumin treatment increased apoptotic cell rate compared to the control group in both CD44^-^ and CD44^+^ PC3 cells (p<0.001). curcumin 15 and 30 µM increased apoptotic rate in CD44^-^ PC3 cells by 30.15±4.03% and 39.72±1.61%, respectively. In CD44^+^ PC3 cells, 50 and 80 µM concentrations of curcumin significantly increased the apoptotic rate by 34.57±1.30% and 43.86±1.52%, respectively (Figure 5c and 5d, p<0.05).

**Curcumin promoted the expression of miR-383-5p and miR-708-5p while downregulating their target genes**

To find possible target genes for miR-383-5p and miR-708-5p, we employed the bioinformatic algorithms miRbase and Target scan. We validated *LDHA* and *PRDX3* as probable targets of miR-383-5p and *RAP1B* and *LSD1* as potential targets of miR-708-5p based on our *in-silico* study.

**Figure 6:**
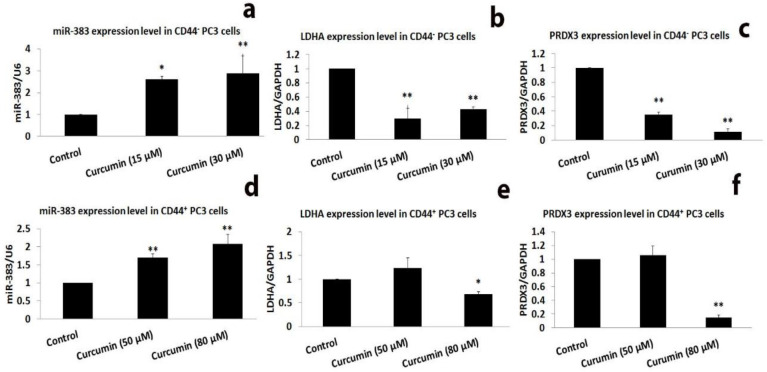
Curcumin induced the expression of miR-383 while suppressing the expression of *LDHA* and *PRDX3* in CD44^±^ PC3 cells. The CD44^±^ PC3 cells were exposed to the subtoxic concentrations of curcumin according to IC_50_ values. qRT-PCR was used for the detection of miRNA and gene expression levels after curcumin treatment for 24 hr. All tests are performed three times. *p<0.05 and **p<0.01

We found that curcumin significantly increases the expressions of miR-383-5p in both CD44^+^ and CD44^-^ prostate cancer cells (Figure 6a and 6d). We used real-time PCR to investigate whether curcumin could silence target genes of miR-383-5p in the CD44^±^ PC3 cells. Curcumin significantly suppressed *LDHA* and *PRDX3* (downstream targets of miR-383-5p) in CD44^-^ cells (Figure 6b and 6c). Curcumin (80 μM) significantly decreased the expression of *LDHA* and *PRDX3* in the CD44^+^ cells, although 50 μM curcumin had no discernible effect on their levels (Figure 6e and 6f). 

**Figure 7:**
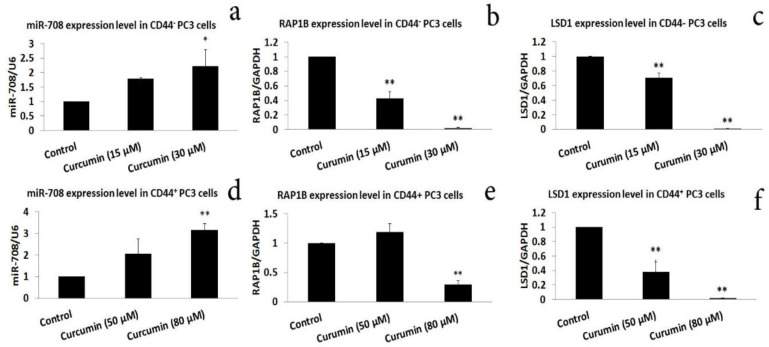
Curcumin induced the expression of miR-708-5p while suppressing the expression of *RAP1B* and *LSD1* in CD44^±^ PC3 cells. The cells were exposed to a subtoxic concentration of curcumin. qRT-PCR was used for the detection of miRNA and gene expression levels after curcumin treatment for 24 hr. All tests were performed three times. *p <0.05 and **p <0.01

We then detected the overexpression of miR-708-5p in the CD44^+^ PC3 cells after treatment with curcumin (Figure 7a and 7d). Curcumin (15 and 30 μM) significantly decreased the expression of *RAP1B* and *LSD1* in CD44-negative cells (p<0.05, Figure 7b and 7c). In CD44^+^ cells, 80 μM curcumin significantly inhibited the expression of *RAP1B* and *LSD1*. But only *LSD1* was suppressed by 50 μM curcumin, and the change in *RAP1B* expression was statistically insignificant (Figure 7e and 7f).

## Discussion

Tumor resistance and recurrence often occur because the majority of conventional cancer therapies, such as chemotherapeutic agents and radiation, are unable to eradicate CSCs (Korkaya et al., 2009; Shafee et al., 2008). There are a variety of cytotoxic effects that curcumin exerts on CSCs. This is caused by a variety of features, including the inhibition of cytokine release, impacts at various points along the pathways for CSC self-renewal, and specific microRNAs that are dysregulated in CSCs (Li and Zhang, 2014; Sordillo and Helson, 2015).

Recent studies assessed the potential effect of curcumin on CSCs when used alone or in conjunction with other anticancer medications. Furthermore, multiple clinical trials have amply shown the safety and tolerability of curcumin (Li and Zhang, 2014). Here, we found that curcumin exerted its cytotoxic effects against both prostate CD44^+^ CSCs and CD44^-^ non-CSCs through induction of apoptosis in a dose-dependent manner. Also, our results revealed that CD44^+^ cells were more resistant to curcumin than CD44^-^ cells. Similar to our findings, Zhang et al. revealed that curcumin suppressed proliferative and invasive characteristics of human prostate CSCs through the induction of expression of specific miRNAs in the DLK1-DIO3 miRNA cluster (Zhang et al., 2018a). Also, Liu et al. showed that curcumin treatment arrested the cell cycle and inhibited proliferation and metastasis in human prostate CSCs. They proposed that curcumin upregulates miR-145 and decreases proliferative characteristics of human prostate CSCs by inhibiting Oct4 expression (Liu et al., 2017b).

Our results also showed that curcumin significantly increased nuclear shrinkage, chromatin condensation, and pycnotic body formation in both CD44^-^ and CD44^+^ PC3 cells in a dose-dependent manner. Consistent with our study, Zhang et al. observed apoptotic bodies and fragmented nuclei in curcumin-treated human glioma CHME cells (Zhang et al., 2018b). In another study, Kantara et al. showed that curcumin decreased the total number of tumorospheres and activated caspase-3 (Kantara et al., 2014).

According to previous studies, curcumin inhibits the growth and invasion of human prostate CSCs *in vitro* by modifying certain miRNAs in the DLK1-DIO3 imprinted gene cluster, such as miR-770-5p and miR-1247 (Zhang et al., 2018a). Additionally, curcumin (10 M) was capable of reducing the cell population with elevated levels of ALDH1, a hallmark specific for breast CSCs, from 7.3% to 1.5% (Ginestier et al., 2007; Kakarala et al., 2010). According to Zhuang et al., curcumin caused glioma CSCs to differentiate and downregulated their capacity for self-renewal (Zhuang et al., 2012). Additionally, while having various effects on CSCs, curcumin is not cytotoxic to normal stem cells (Sordillo and Helson, 2015).

In this study, we used curcumin as an attempt to induce the expression of tumor suppressor miRNAs, miR-708-5p and miR-383-5p in the human prostate CD44^+^ CSCs and CD44^- ^non-CSCs. Our results showed that curcumin significantly increased the expression level of miR-708-5p in both CD44^+^ and CD44^-^ cells in a dose-dependent manner. Similar to our study, many attempts have been made to induce the expression of miR-708 in different cancers. In 2012, miR-708 was first introduced as a therapeutic target of PC by Saini et al. They have revealed that induction of miR-708 expression in CD44^+^ PC cells suppressed tumorigenicity. In contrast, the knockdown of miR-708 increased tumor growth in CD44^-^ PC cells (Saini et al., 2012). The therapeutic roles of miR-708 also have been confirmed in many cancers including prostate, breast, cervical and pancreatic carcinoma (Huang et al., 2019; Ma et al., 2016; Zou et al., 2020). The low expression of miR-708 is involved in the neuroendocrine differentiation of PC, which causes a poor prognosis in patients. In 2019, Shan et al. found that the overexpression of EZH2 inhibits miR-708. They also found that induction of miR-708 reduces the number of CD44^+^ neuroendocrine PC cells (Shan et al., 2019). 

Also, our results showed that curcumin suppressed the expression of *RAP1B* and *LSD1*, two target genes of miR-708, which are also crucial in the pathogenesis of PC. Previous studies have shown that *LSD1* suppresses PC cell viability (Gao et al., 2020). Similar to our findings, Ma et al. demonstrated that suppression of *LSD1* might phenocopy the effect of miR-708 overexpression in MDA-MB-231 cells. Amplification of *LSD1* may offset miR-708's effects on invasion and proliferation. and the findings suggest that miR-708 may have a tumor suppressor gene role in the emergence of breast cancer (Ma et al., 2016). Furthermore, *RAP1B* expression is correlated with the immune infiltration of tumors in various cancers (Cui et al., 2021). In a similar study, Lin et al. showed that glucocorticoids reduce invasive characteristics of ovarian cancer by inducing the expression of miR-708 and inhibiting *RAP1B* (Lin et al., 2015). 

Since CD44 is a hyaluronic acid receptor, it has a crucial role in the adhesion and migration of cancerous cells. At first, Bucay et al. showed that miR-383-5p, which is highly downregulated in PC, could be essential for PC metastasis and stemness by directly targeting CD44. The restoration of miR-383 expression in PC cells has proven anti-metastatic and anti-tumorigenic effects (Bucay et al., 2017). 

Our results revealed that curcumin significantly increased the expression levels of miR-383-5p in both CD44^+^ and CD44^-^ cells. In a similar study, Lv et al. showed that allicin, a phytochemical extracted from garlic, induces miR-383 expression in gastric cancer. Allicin targets the miR-383/ERBB4 axis to inhibit the invasion of gastric cancer (Lv et al., 2020). A recent study conducted by Huang et al. also approved that induction of miR-383 expression could inhibit growth and metastasis in PC (Huang et al., 2021). Conversely, downregulation of miR-383 by TMPO-AS1 in lung adenocarcinoma and pancreatic carcinoma and MIR4435-2HG in head and neck squamous cell carcinoma led to tumor progression (Mu et al., 2020; Wang et al., 2021; Xue et al., 2021).

Although tumor suppressive roles of miR-383 have been confirmed in various cancers such as malignant melanoma and glioma, it was shown that high miR-383 expression in cholangiocarcinoma is associated with advanced tumor stages, metastasis, and poor prognosis (Wan et al., 2018; Xu et al., 2021a; Xu et al., 2021b).

Our results also confirmed the downregulation of *PRDX3* and *LDHA*, target genes of miR-383, by curcumin in CD44^±^ PC cells. *PRDX3* and *LDHA* are typically overexpressed in PC. *PRDX3*, acting as an antioxidant, protects PC cells from reactive oxygen species and promotes the survival of cancerous cells (Whitaker et al., 2013). *LDHA* plays a vital role in the Warburg effect (aerobic glycolysis) which provides a sufficient number of metabolites for the rapid growth of cancerous cells. Vieira et al. showed that overexpression of *LDHA* in the clinical specimen of PC patients is associated with therapeutic resistance (Vieira et al., 2021). In another study, the MYC-LDHA axis was shown to be important in PC pathogenesis, and FAM46B by targeting this axis, induces apoptosis and inhibits glycolysis in PC cells (Liang et al., 2020). Altogether, suppression of expression of *PRDX3* and *LDHA* is another aspect of the promising roles of curcumin in PC treatment.

Altogether, our study revealed that curcumin suppressed cellular proliferation and induced apoptosis in CD44^+^ and CD44^-^ PC cells. We also showed that curcumin, by promoting the expression of miR-383 and miR-708 and inhibiting their target genes, induced its cytotoxicity against CD44^±^ PC cells. Considering that traditional cancer chemotherapeutic agents and radiotherapy cannot eradicate CSCs, the cytotoxicity of curcumin against CD44^+^ human prostate CSCs is a remarkable feature. We trust that curcumin could be established as a promising adjuvant to current PC treatment options following research in clinical settings.

## Conflicts of interest

The authors declare that they have no conflict of interest
